# Mapping of common bunt resistance gene *Bt9* in wheat

**DOI:** 10.1007/s00122-017-2868-6

**Published:** 2017-02-25

**Authors:** Philipp Matthias Steffan, Anna Maria Torp, Anders Borgen, Gunter Backes, Søren K. Rasmussen

**Affiliations:** 1 Department of Plant and Environmental Sciences, University of Copenhagen, Thorvaldsensvej 40, 1871 Frederiksberg, Denmark; 2 KWS LOCHOW GMBH, Ferdinand-von-Lochow-Straße 5, 29303 Mons, Germany; 3 Department of Organic Agricultural Sciences, University of Kassel, Nordbahnhofstraße 1a, 37213 Witzenhausen, Germany; 4 Agrologica, Houvej 55, 9550 Mariager, Denmark

## Abstract

**Key message:**

The *Bt9* resistance locus was mapped and shown to be distinct from the *Bt10* locus. New markers linked to *Bt9* have been identified and may be used to breed for resistance towards the seed-borne disease.

**Abstract:**

Increasing organic wheat production in Denmark, and in other wheat-producing areas, in conjunction with legal requirements for organic seed production, may potentially lead to a rise in common bunt occurrence. As systemic pesticides are not used in organic farming, organic wheat production systems may benefit from genetic resistances. However, little is known about the underlying genetic mechanisms and locations of the resistance factors for common bunt resistance in wheat. A double haploid (DH) population segregating for common bunt resistance was used to identify the chromosomal location of common bunt resistance gene *Bt9*. DH lines were phenotyped in three environments and genotyped with DArTseq and SSR markers. The total length of the resulting linkage map was 2882 cM distributed across all 21 wheat chromosomes. *Bt9* was mapped to the distal end of chromosome 6DL. Since wheat common bunt resistance gene *Bt10* is also located on chromosome 6D, the possibility of their co-location was investigated. A comparison of marker sequences linked to *Bt9* and *Bt10* on physical maps of chromosome 6D confirmed that *Bt9* and *Bt10* are two distinct resistance factors located at the distal (6DL) and proximal (6DS) end, respectively, of chromosome 6D. Five new SSR markers *Xgpw4005-1, Xgpw7433, Xwmc773, Xgpw7303* and *Xgpw362* and many SNP and PAV markers flanking the *Bt9* resistance locus were identified and they may be used in the future for marker-assisted selection.

**Electronic supplementary material:**

The online version of this article (doi:10.1007/s00122-017-2868-6) contains supplementary material, which is available to authorised users.

## Introduction

Although common bunt [caused by *Tilletia caries* (DC.) Tul. & C. Tul (syn. *T. tritici* (Bjerk.) G. Winter in Rabenh.) and *T. foetida* (Wallr.) Liro (syn. *T. laevis* Kühn in Rabenh.)] is a major disease in wheat (*Triticum aestivum* L. subsp. aestivum; van Slageren 1994) worldwide, it has received surprisingly little attention in the last 50 years. The ease of control of common bunt infection in wheat by the application of seed treatments with systemic fungicides (based on hexachlorobenzene, carboxin, difenoconazole or tebuconazole; Hoffmann and Waldher 1981) might be one reason for the lack of research and knowledge about wheat–pathogen (host–common bunt) interactions.

Growing concern about the environmental impact of agricultural production and increased organic wheat production (Eurostat 2014) demands a search for alternative modes of control. Furthermore, in many parts of the world, farmers lack access to fungicides, and common bunt has been a continuous threat to wheat production (e.g. Mamluk 1998). The use of host resistance genes in wheat breeding offers a mode of control of common bunt infection.

Sixteen resistance genes, designated *Bt1*–*Bt15* and *Btp*, have been identified (Goates 2012) and further resistance sources among Ukrainian and Russian germplasm (Martynov and Dobrotvorskaya 2003) and gene bank accessions (Goates and Bockelman 2012) have been reported. In addition, introgressions of common bunt resistance factors from rye (*Secale cereale*, Martynov and Dobrotvorskaya 2003), triticale (Ciuca 2011), barley (*Hordeum vulgare*, Rubiales et al. 2001), *Aegilops glaucum* (Martynov and Dobrotvorskaya 2003), *Ae. cylindrica* (Galaev et al. 2006), *Ae. ventricosa* (Babayants et al. 2006), *Triticum erebuni* (Babayants et al. 2006) and *Agropyron intermedium* (Goates 1996) have been reported.

To the authors’ knowledge, the genetic locations of only three Bt genes and 15 quantitative resistance factors (quantitative trait loci, QTL) for common bunt have so far been mapped (Table 1). In addition, locations of the following Bt genes have been suggested: *Bt5* (R.J. Metzger and C.W. Scheller, pers. comm, cited in McIntosh et al. 1998) and *Bt6* (ref. 1005 in McIntosh et al. 1998, but not retrievable) on chromosome 1B, *Bt7* on chromosome 2D (R.J. Metzger, pers. comm, cited in McIntosh et al. 1998), and *Bt11* on chromosome 3B (Ciuca 2011). Although *Bt8* has not been mapped, it is not located on chromosomes 5A, 1B or 2D (Waud and Metzger 1970).

**Table 1:** Bt genes and QTL for common bunt resistance in wheat for which chromosomal locations are known

Gene	Chromosome	References
*Bt1*	2B	Sears et al. (1960); Gupta (2007)
*Bt4*	1B	Schmidt et al. (1969)
*Bt10*	6DS	Menzies et al. (2006)
*QCbt.crc-1B.1*	1BS	Fofana et al. (2008)
*QCbt.crc-1B.2*	1BL	Fofana et al. (2008)
*Xgwm 374* ^a^	1BS	Wang et al. (2009)
*QCbt.spa-1B*	1B	Singh et al. (2016)
*Xgwm273* ^a^	1B	Dumalasová et al. (2012)
*QCbt.spa-4B*	4B	Singh et al. (2016)
*QCbt.spa-4D*	4D	Singh et al. (2016)
*Xgwm408* ^a^	5B	Dumalasová et al. (2012)
*QCbt.spa-5B*	5B	Singh et al. (2016)
*QCbt.spa-6D*	6D	Singh et al. (2016)
*QCbt.crc-7A*	7AL	Fofana et al. (2008)
*Xpsp3050* ^ a ^	7A	Dumalasová et al. (2012)
*Xgwm43* ^ a ^	7B	Dumalasová et al. (2012)
*QCbt.spa-7B.1*	7B	Knox et al. (2013)
*QCbt.spa-7D*	7D	Singh et al. (2016)

^a^For QTLs that were not designated in accordance with McIntosh et al. (1998), the name of the nearest flanking marker is given

*Tilletia* sp. and wheat follow the classic gene-for-gene concept of pathogen–host interactions (Reed 1928; Bressman 1931). It has been shown numerous times that such resistance may easily be overcome by the pathogen (e.g. leaf rust, Long et al. 1998). In fact before the introduction of hexachlorobenzene in 1956 for seed treatment to control common bunt, a resistance breakdown was noticed (Hoffmann 1971). The threshold level for common bunt is very low, because the disease is not only affecting the yield but indeed also affect quality of the grain at a much lower infection level. It is possible that a sum of several additive partial resistances could accumulate to sufficient resistance level, but so far this has not been documented.

Marker-assisted selection in plant breeding offers the possibility to breed lines with more than one resistance source (Kloppers and Pretorius 1997), and thus create host resistance diversity that may be more difficult to overcome by pathogens and provide more durable resistance. However, marker-assisted selection and gene-pyramiding strategies rely on sound genetic understanding of resistance factors.

The early identification of chromosomal locations of resistance factors was achieved by the use of monosomic wheat lines (Sears et al. 1960; Schmidt et al. 1969), while more recently molecular markers have been employed (e.g. Demeke et al. 1996; Laroche et al. 2000; Ciucã 2011; Singh et al. 2016). Marker-assisted selection for common bunt resistance in wheat is, however, only applied for the *Bt10* resistance gene (Menzies et al. 2006). Common bunt resistance genes *Bt9* and *Bt10* were derived from the same cross between common bunt-susceptible *cv*. Elgin and gene bank accession PI 178383, a landrace collected in Turkey (Harlan 1950). From this cross, gene bank accessions PI 554099 and PI 554118 were developed, carrying resistance genes *Bt9* and *Bt10*, respectively (Goates 1996). Their classification into two distinct resistance factors has so far relied on their different resistance reactions to various common bunt isolates (e.g. Goates 2012) and, to the authors’ knowledge, no genetic evidence about their distinctness is available. In order to advance molecular resistance breeding in wheat against common bunt, this study (1) assessed the efficacy of the resistance gene *Bt9* in Denmark, (2) used the offspring of a cross between PI 554099, carrying *Bt9* (Goates 2012), and a common bunt-susceptible cultivar to map the chromosomal location of *Bt9*, and (3) investigated the possible co-location of resistance genes *Bt9* and *Bt10*.

## Materials and methods

### Plant material

A population of 91 double haploid (DH) lines segregating for common bunt resistance gene *Bt9* was generated (Erik Tybirk, Nordic Seed A/S, Galten, Denmark) from microspores off approximately 15 F1 plants following a proprietary protocol. The cross was made between wheat accession PI 554099 (National Small Grains Collection, Aberdeen, Idaho, USA), carrying resistance gene *Bt9*, and common bunt susceptible-wheat cv. Cortez (Wiersum Plant Breeding, Winschoten, The Netherlands).

### Fungal spores

Common bunt teliospores were received from Bent J. Nielsen (Aarhus University, Aarhus, Denmark) as a bulk composite from a broad selection of locations in Denmark, representing the virulence spectrum of Danish common bunt isolates. The bulk composite of common bunt spores was maintained by inoculating a broad range of common bunt-susceptible wheat accessions which have been shown not to possess any common bunt resistances. For a specific study against a single gene, in this case *Bt9*, it makes no difference which race is used or if it is a mixture of races, as long as none of the races in a bulk has virulence against the gene in question. It has been documented in our association mapping that the bulk of spores did not contain any virulence against *Bt9* (Steffan et al., unpublished data; Borgen 2015).

### Phenotyping reaction to common bunt

The DH population was evaluated for common bunt resistance under field conditions in 2012 and 2013 in Mariager (56.39°N 10.01°E), Denmark, and in 2013 under greenhouse conditions at the experimental farm (55.67°N; 12.30°E) in Taastrup, Denmark. The environments were designated Fd12 and Fd13 for the field assessments in 2012 and 2013, respectively, and Gh13 for the greenhouse assessment in 2013. DH lines were assessed in two replications in Fd12 and in one replication in Fd13 and Gh13.

Sowing of field trials Fd12 and Fd13 was performed as follows: 50–80 seeds of each DH line per replicate were sown by hand in 1-m rows in the field in mid-October in 2011 and in late October in 2012. Seeds were inoculated with common bunt by mixing and shaking with an abundance of bunt teliospores in a container, and sieved in a mesh allowing spores to pass but retaining the wheat seeds prior to sowing. For the sowing of the greenhouse assessment Gh13, 50 seeds per DH line were sown in plastic containers filled with potting soil in October 2012. Temperatures were the standard of the greenhouse with 18 and 13 °C during the day and night, respectively, somewhat higher temperature than recommended (Borgen and Kristensen 2003). From 4 weeks after sowing, plants were vernalised for 8 weeks at 6 °C in dim light.

### Statistical analysis

Common bunt resistance reaction in DH lines was scored as the percentage of wheat spikes with at least one spikelet with bunt sori relative to total number of spikes. An analysis of variance based on a linear model was used to estimate the effects of genotypes, environments and their interaction on common bunt resistance reactions.

A mixed effects model was used to extract best linear unbiased predictions (BLUPs) for each DH line:

1 $${y_{{\text{d,r,e}}}} = \mu + E + (G) + \varepsilon ,$$

where the common bunt score *y* of DH line *d* in replication *r* in environment *e* is given by *y*_*d,r,e*_ and the overall mean is indicated by *µ*. The effect of the three environments Fd12, Fd13 and Gh13 is included as a fixed effect *E*, and the genotype effect is included as the random effect (*G*). The error term is denoted *ε*.

### Genotyping using DNA markers

DNA extraction was carried out as described in Orabi et al. (2014). Genotyping with DArTseq markers was performed by Triticarte Pty. Ltd (Canberra, Australia). DArTseq is a marker technique built on DNA complexity reduction as described for DArT markers (Akbari et al. 2006), followed by sequencing of the DNA representations on next-generation sequencing platforms. The method generates a high number of SNP (single-nucleotide polymorphism) and PAV (presence and absence variant) markers that can be used for, e.g. genetic mapping (Cruz et al. 2013; Li et al. 2015). To investigate whether *Bt9* and *Bt10* co-locate, 41 SSR markers known to span chromosome 6D were selected from genetic and physical maps available at Graingenes (http://wheat.pw.usda.gov/GG3/) and cMAP (https://ccg.murdoch.edu.au/cmap/ccg-live/cgi-bin/cmap/viewer). Out of the 41 SSR primer pairs tested, 20 resulted in the amplification of a total of 29 polymorphic loci that could be mapped in the DH population. Primer sequences and chromosomal location for the polymorphic markers are shown in Supplementary Table S1. PCR amplification of SSR markers was carried out according to Orabi et al. (2014). SSR fragments were analysed by capillary electrophoresis using an AB/Hitachi 3130xl Genetic Analyzer (Thermo Fisher Scientific Inc., MA, USA), and allele sizes were determined using the software GeneMarker v. 1.95 (SoftGenetics LLC, State College, PA, USA). In addition to this, the PCR marker FSD_RSA linked to the *Bt10* bunt resistance gene (Laroche et al. 2000) was used on the two parent lines of the mapping population to test for the presence/absence of this gene. Wheat accessions PI 554118 (*Bt10*) and PI 178383 (*Bt8, Bt9, Bt10*) were used as positive controls for the *Bt10* gene, while PI 209794 (*Bt0*) was used as the negative control. PCR was carried out in 10 µl reactions containing 1× Key buffer (VWR International), 0.2 µM of each dNTP, 0.25 µM of each primer (FSD and RSA), 0.25 U VWR Taq polymerase and 40 ng of wheat DNA. Primer sequences were obtained from Laroche et al. (2000) and are provided in Table S1. PCR reactions were carried out on a Verity PCR machine (Applied Biosystems) under the following conditions: initial denaturation at 94 °C for 2 min, 35 cycles of 94 °C for 1 min, 44 °C for 1 min, and 72 °C for 2 min, followed by a final extension step at 72 °C for 7 min. PCR products were analysed on a 1.5% agarose gel (140 V for 40 min).

### Map construction and QTL analysis

Before further analysis, SNP and PAV markers with more than 5% heterozygous scores were removed from the dataset. As the DH population was developed from several F1, a total of 2274 co-dominant SNPs and 31 SSR loci were used to check the DH lines for signs of heterogeneity. The vast majority of the DH lines were homozygotic showing heterogeneity for less than 1% of these loci, while three DH lines were identified with more that 5% heterozygous scores for co-dominant SNPs and/or signs of heterozygosity in some of the SSR loci and were removed from further analysis. Heterozygous scores for markers with less than 5% of this type of score were changed to missing. Subsequently markers with a total of more than 10% missing data points and/or significant distorted segregation (*P* < 0.001) were removed from the dataset. The remaining SNP, PAV and SSR markers were analysed using the bin functionality of the QTL IciMapping program (Meng et al. 2015) to identify redundant markers. During binning, redundant markers were deleted by missing rate leaving those with fewest missing data points as representatives of the bin. The remaining markers were used for grouping and map calculation in JoinMap^®^ 4.1. (Van Ooijen 2006). Grouping was initially carried out at a LOD threshold of 8–12. Evaluation and chromosome assignment of groups were carried out based on information obtained from the wheat DArTseq map described by Li et al. (2015), as well as from a blast search of SNP and PAV sequences against a local database of wheat sequences based on the Triticum_aestivum.IWGSC1+popseq.31 genome assembly downloaded from Ensemble plants (http://plants.ensembl.org/Triticum_aestivum/Info/Index) in April 2016. Only markers with a unique location, maximum alignment length and not more than 1 mismatch were used during assignment of linkage groups. Linkage maps were calculated using the regression mapping algorithm in JoinMap^®^ 4.1 with the Kosambi mapping function for the calculation of map distances.

Inclusive composite interval mapping (ICIM) (Li et al. 2007) implemented in the QTL IciMapping software was used to scan linkage groups for QTL. The QTL analysis was carried out for each environment (Fd12, Fd13 and Gh13) separately as well as on BLUPs derived from model (1) as representatives of the genotypic effect of each DH line across the three environments. These BLUPS are designated Bunt-3Env. The significance levels for declaring a QTL significant were obtained as the 99th percentile of the maximum LOD scores derived from 5000 permutations, and were determined to be 4.2, 4.2, 4.5 and 4.0 for Bunt-3Env, Fd12, Fd13 and Gh13, respectively. Mapchart 2.3 (Voorrips 2002) and Sigmaplot 13.0 were used to draw figures.

For linkage group(s) with significant QTL, the physical position of relevant SNP and PAV markers was obtained (when available) from a BLAST search against the Triticum_aestivum.IWGSC1+popseq.31 genome assembly hosted at Ensemble plants (http://plants.ensembl.org/Triticum_aestivum/Info/Index). All hits used had an *E* value better than 1E−20. The sequences and physical location of the SSR markers were obtained from the integrated physical and genetic maps of the wheat D genome (Jia et al. 2013) available at https://ccg.murdoch.edu.au/cmap/ccg-live/ and/or from BLAST of the primer sequences against the Triticum_aestivum.IWGSC1+popseq.31 genome assembly. Retrieved sequences were confirmed by searching for the presence of both primer sequences as well as the expected SSR repeat. Sequences of DArT markers used for the comparison of maps were retrieved from the IWGSC survey sequence annotation viewer hosted at https://urgi.versailles.inra.fr/gb2/gbrowse/wheat_survey_sequence_annotation/. Finally, the genetic map for linkage groups with significant QTL was recalculated using the order on the physical map as fixed order in JoinMap^®^ 4.1.

## Results

### Common bunt resistance scoring

Common bunt infection was successful in all three environments Fd12, Fd13 and Gh13. An analysis of variance indicated a significant influence of genotypes, environments and their interaction on common bunt scores (Table 2), explaining 52, 9 and 29% of the total variation, respectively.

**Table 2:** Analysis of variance of common bunt scores for 88 DH lines tested in three environments

Effect	*DF*	SS	MS	*F* value	*P* value
Environments	2	39783.1	19891.5	137.4	<0.0001
DH lines	87	225688.5	2594.1	17.9	<0.0001
Envir × DH lines	152	125024.9	822.5	5.7	<0.0001
Residual	325	47067.3			

*DF* degrees of freedom, *SS* sum of squares, *MS* mean sum of squares

**Fig. 1:**
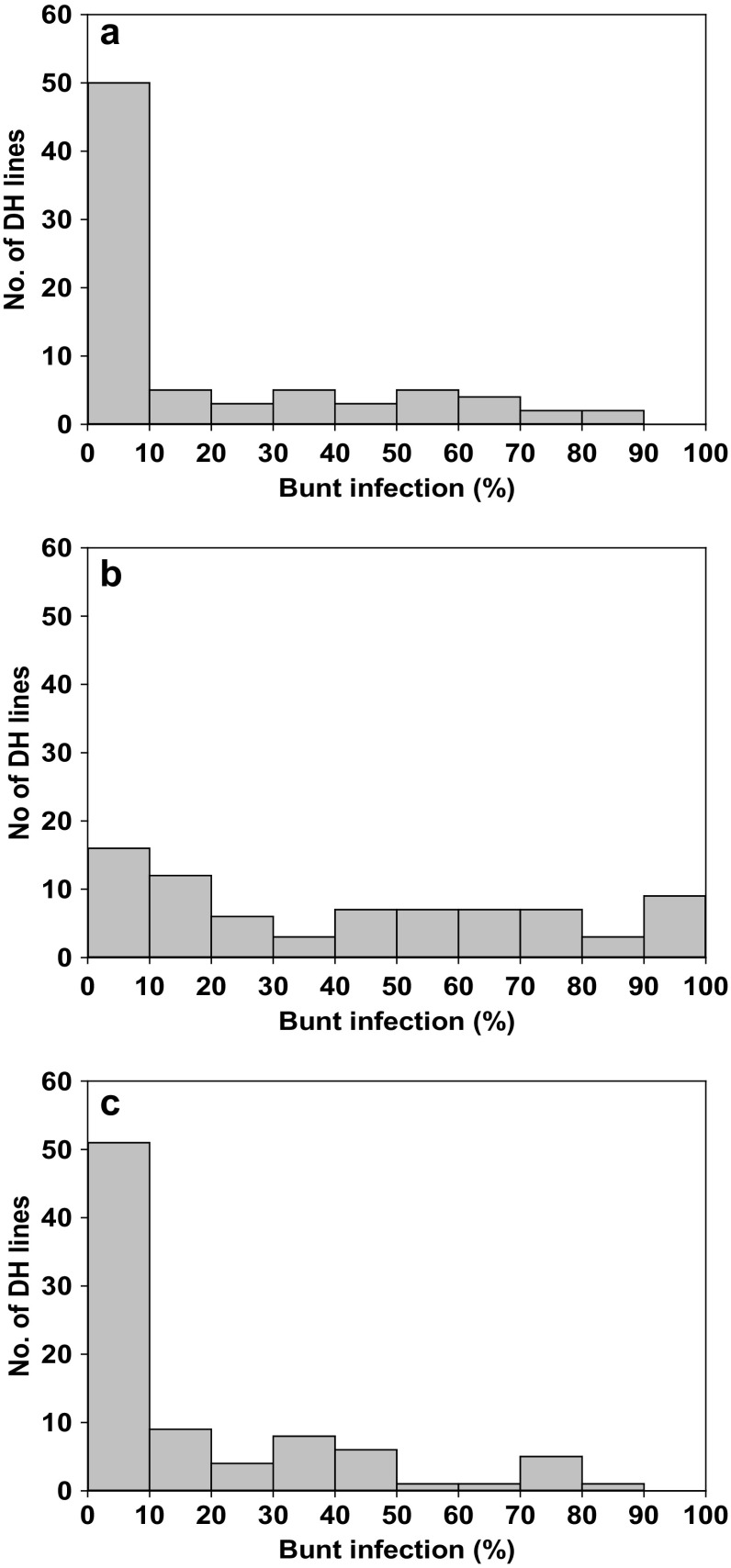
Common bunt assessment scores in DH lines segregating for common bunt resistance gene *Bt9* at environments: **a** Fd12, **b** Fd13 and **c** Gh13. The number of DH lines scored in each environment is given in Table 3

Infection levels of DH lines were similar in Fd12 and Gh13, with average common bunt incidences of 17.2 and 17.0%, while the average incidence was 41.8 in Fd13 (Table 3; Fig. 1). The resistance gene donor, wheat accession PI 554099, did not show any common bunt infection in location Fd12, while an average infestation level of 3.2% were observed in location Gh13 (Table 3). The number of spikes available in wheat accession PI 554099 was too low to be analysed in Fd13. The susceptible parent *cv*. Cortez showed high incidences of common bunt, with high infection levels of 61.0% in location Fd12 and 92.3% in location Fd13, while a markedly lower infection of 6.7% were observed in location Gh13 (Table 3).

**Table 3:** Range of infection levels, average infection and standard deviation of infection levels in DH lines segregating for common bunt resistance gene *Bt9* in individual environments (Fd12, Fd13, Gh13 as well as for the BLUPs calculated across the three environments (Bunt-3Env)

Environment	No. DH lines	No. spikes	Range	Mean	Std dev	PI 554 099	*cv*. Cortez
Bunt-3Env*	88	13,451	-18.4–50.5	0	17.5	-14.3	11.6
Fd12	79	4610	0–84.7	17.2	24.9	0	61.0
Fd13	77	1231	0–100	41.8	33.3	n.a.	92.3
Gh13	86	7610	0-88.5	17.0	22.6	3.2	6.7

The last two columns indicate the parental scores

*PI 554099* resistance gene donor, *cv. Cortez* susceptible parent

*BLUPs from Model (1)

### Map construction and QTL analysis

Genotyping of the DH lines with DArTseq markers yielded 3129 polymorphic SNP markers and 8109 polymorphic PAV markers. The final map calculated using JoinMap^®^ 4.1 consisted of 34 linkage groups, of which all could be assigned to one of the 21 wheat chromosomes based on information from the wheat DArTseq consensus map described by Li et al. (2015) and BLAST against the *Triticum_aestivum*.IWGSC1+popseq.31 genome assembly (Supplementary Table S2). The number of markers with a unique position on the linkage map was 1734, representing a total of 7039 SNP, PAV and SSR markers when the binned markers were counted. The total length of the resulting linkage map was 2882 cM distributed across all 21 chromosomes of hexaploid wheat.

**Fig. 2:**
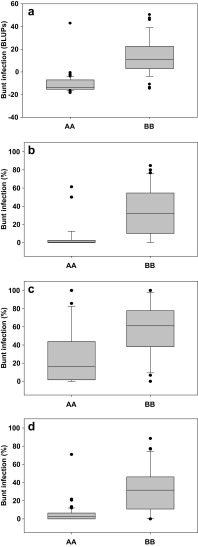
Allelic substitution effect for SSR marker *Xgpw4005-1* linked to *Bt9* on common bunt resistances for **a** Bunt-3Env, **b** Fd12, **c** Fd13 and **d** Gh13. *AA* alleles from resistant parent PI 554099 and *BB* alleles from susceptible parent *cv*. Cortez

Using inclusive composite interval mapping, the presence of a single significant QTL for bunt resistance could be identified at the distal end of wheat chromosome 6DL. The QTL was highly significant in both the individual environments Fd12 (LOD 8.0) and Gh13 (LOD 9.2), as well as when the BLUPs (Bunt-Env3, LOD 13.9) were analysed (Table 4). In location Fd13, a QTL was indicated at position 129 cM; however, the LOD score (4.0) was not significant at the significance level of 4.5 determined after 5000 permutations (99th percentile). The identified QTL explained between 37.7 and 53.7% of the variation (Table 4). Even when the LOD threshold were manually set to LOD = 3.0, there were no indications of any additional QTLs for bunt resistance in other positions of the genome. Wheat accession PI 554099 contributed the allele reducing common bunt infection in DH lines in all cases (Fig. 2). The position of the QTL differed between Fd12, Gh13 and Bunt-3Env, but combining results from the individual analyses suggested that the QTL is most likely positioned somewhere between 124.5 and 132.5 cM on the current map, a region flanked by markers *1022670* and *3022667* (Table 4). Counting the binned markers, a total of 111 SNP, PAV and SSR markers mapped to this interval. These include the SSR markers *Xgpw7433, Xwmc773* and *Xgpw7303*, which all mapped together with *Xgpw4005-1* at position 127 cM on the current map. On the physical map of wheat, this corresponded to a region from approximately 170.5 to 176.5 Mbp, a region that contains around 270 genes on the current assembly of wheat chromosome 6D.

**Table 4:** QTL for bunt resistance gene *Bt9* at two different locations Fd12 and Gh13 as well as for BLUPs calculated across the three environments (Bunt-3Env) identified through inclusive composite interval mapping. Flanking markers (Left and Right markers), LOD score, percent variance explained (PVE), allelic substitution effect as well as left (LeftCI) and right (RightCI) borders of the One-LOD drop of confidence interval for the QTL are provided

Environment	Chromosome	Position (cM)	Left marker	Right marker	LOD	PVE%	Allelic effect	Left CI	Right CI
Bunt-3Env*	6D	127	Xgpw4005-1	3024256	13.9	52.7	12.8	126.5	127.5
Fd12	6D	132	1040566	3022667	8.0	37.7	15.3	129.5	132.5
Gh13	6D	125	1022670	3028756	9.2	39.9	14.3	124.5	125.5

*BLUPs from Model (1)

**Fig. 3:**
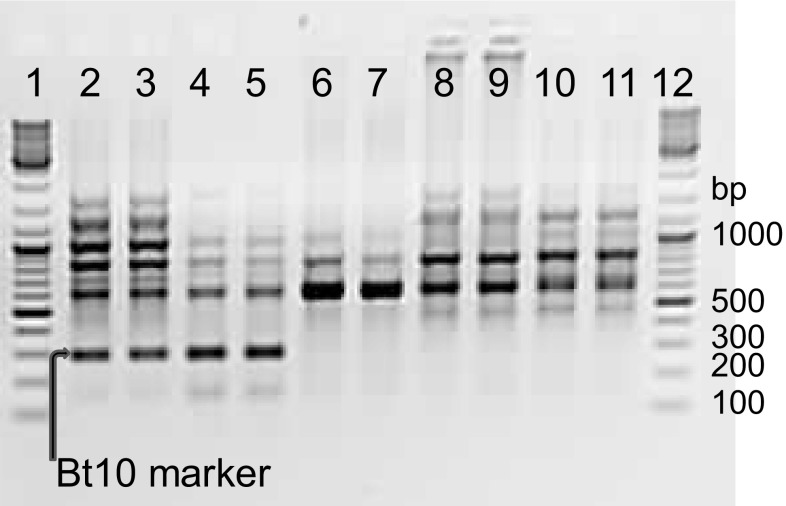
Agarose gel of BT10 PCR with FSD and RSA primers. Lane identification 1,12 = Generuler DNA ladder mix (#SM0331, Thermo Scientific), 2–3 = PI 554118 (*Bt10*), 4–5 = PI 178383 (*Bt8, Bt9, Bt10*), 6–7 = PI 209794 (*Bt0*), 8–9 = PI554099 (*Bt9*) and 10–11 = cv Cortez

**Fig. 4:**
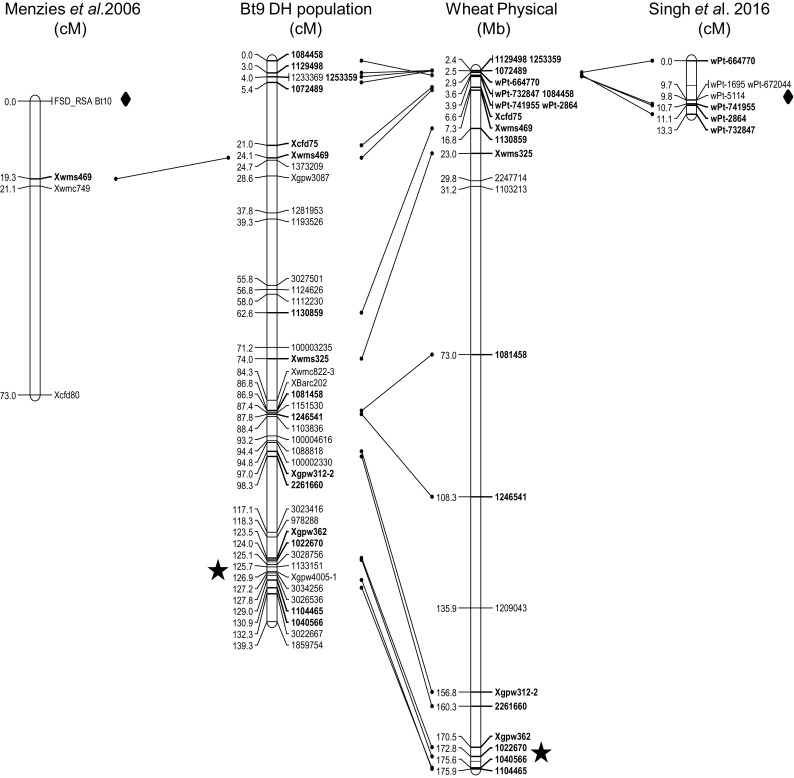
Genetic and physical map of chromosome 6D of wheat, showing the position of the *Bt9* region. The genetic map was created using the 88 DH lines from the population PI 554099 × *cv* Cortez, while the physical map was created based on the Triticum_aestivum.IWGSC1+popseq.31 genome assembly. On these two maps only one marker is shown per bin (unique positions). Markers with prefix Xgpw, Xwms, Xwmc, Xcfd or Xbarc are SSR markers, while markers without a prefix are DArTseq markers. The position of the *Bt10* region is indicated by interpolation from two previous genetic maps developed by Menzies et al. (2006) and Singh et al. (2016). The *Bt9* region is indicated by a *star*, while the *Bt10* region is indicated by a *square*

As expected, amplification of the FSD_RSA marker linked to the *Bt10* resistance gene resulted in a ~275 bp long amplification product in the two *Bt10* containing accessions PI 554118 (*Bt10*) and PI 178383 (*Bt8, Bt9, Bt10*), while no product of the expected length was seen in the negative control PI 209794 (*Bt0*). None of the parents of the mapping population used in the present study showed any sign of the Bt10 amplification product (Fig. 3); therefore, it was not possible to map *Bt10* directly in the DH population. However, the two SSR markers *Xwmc749* and *Xwms469* known to map 19–20 cM below the *Bt10* gene (Menzies et al. 2006) mapped to the same bin at position 24.1 cM in this population and located to a physical position around 7.2–7.6 Mbp on the wheat genome assembly (Fig. 4). In addition, the DArT markers *wPt-741955* and *wPt-2864* that mapped very close to *QCbt-spa-6D*, probably corresponding to *Bt10* (Singh et al. 2016), were found to be located at a physical position of approximately 3.9 Mbp. Thus, interpolation between these maps indicates that the *Bt10* gene locates to a position in the gap at 5.4 to 21.0 cM between markers *1072489* and *Xcfd75* on the map developed during this study (Fig. 4).

## Discussion

In this study, common bunt resistance was assessed in wheat DH population in three environments, and by the use of markers a QTL was identified at the distal end of chromosome 6DL explaining a high percentage of the observed phenotype variability, and with a strong allelic substitution effect.

The identification of a single large-effect resistance QTL in the present population was not unexpected since PI 554099 is known to carry the *Bt9* gene and *cv* Cortez has been shown to be highly susceptible to common bunt. However, the presence of additional minor effect QTL in the population cannot at present be completely excluded, since the distribution of DArTseq markers was uneven (Table S2), resulting in poor coverage of some chromosomes. The total number of markers was much lower on the D genome (1088) compared to the A (3106) and B (2845) genomes (Table S2), a phenomenon that was also observed by Li et al. (2015) in a mapping study using wheat DArTseq markers. In the case of the present study, however, the shortest linkage groups were observed on chromosomes 6B (48 cM) and 7B (73 cM). Progress in wheat genome sequencing (Brenchley et al. 2012) and new marker techniques with a better coverage of the wheat genome (Wilkinson et al. 2012) may close the remaining gaps in the not-too-distant future. The coverage of chromosome 6D, where both the *Bt9* and *Bt10* genes for bunt resistance map, was generally good, with a genetic map length of 139 cM and a physical map covering the region from 2.4 to 175.9 Mb. The total length of the wheat 6D assembly is at present 177.0 Mb (http://plants.ensembl.org/Triticum_aestivum/Info/Index). The large gap in the physical map is likely to span the centromeric region as *Xwms325* has been located above the centromere and *XBarc202* has been located to bin 6DL1-0.47-0.68 on 6DL (http://wheat.pw.usda.gov/GG3/). Restricted recombination in centromeric regions has been observed on, e.g. wheat chromosome 3B (Choulet et al. 2014) and is a likely cause of the large gap seen on the physical map here.

To the authors’ knowledge, no genetic evidence about the distinctness of bunt resistance genes *Bt9* and *Bt10* was available prior to this study. In contrast, in a compressed mixed model association mapping study in which wheat accessions were grouped according to genetic relatedness based on genotyping with DArT markers, wheat accessions PI 178383 (*Bt8, Bt9, Bt10*), PI 554099 (*Bt9*) and PI 554118 (*Bt10*) formed a distinct group within 248 wheat accessions (Steffan et al., unpublished data). However, it is believed that the localisation of *Bt9* at the distal end of chromosome 6DL and the genetic and physical distance of more than 100 cM and approximately 170 Mb, respectively, between *Bt10* located at the proximal end of chromosome 6DS (Menzies et al. 2006; Singh et al. 2016) and *Bt9* on the map presented here (Fig. 4) provide strong evidence that *Bt10* and *Bt9* are two distinct resistance factors, and confirm the previously used classification based on phenotypic evaluations.

Common bunt resistance gene *Bt9* was shown to be an effective source of resistance to common bunt in Denmark. No infection was observed in PI 554099 under field conditions neither in this study (2012) nor in another study conducted at the same nursery in Mariager during 2011 and 2012 (Steffan et al., unpublished data, Borgen 2015). However, low levels of infection could be observed in wheat accession PI 554099 under greenhouse conditions during this study. Inconsistent results of common bunt resistance tests under greenhouse conditions have previously been reported (Schmidt et al. 1969). However, since average infection levels of DH lines under greenhouse and field conditions were similar, it was concluded that *Bt9* proved effective under both field and greenhouse conditions.

The failure to detect the *Bt9* QTL in the field screen in 2013 (Fd13) may be attributed to the late sowing in October 2012, which had an effect on the number of plants available for assessment as well as on the overall infection level. Sowing into cooler soil conditions, as was the case in autumn 2012 compared to the sowing in autumn 2011, is known to increase infection levels (Reed 1928; Gaudet and Puchalski 1990), which was also observable in the present study, and may have reduced the effect of the *Bt9* QTL in the field assessments of 2013.

The high number of plants needed and the long time required to assess common bunt resistance reactions phenotypically render the availability of a molecular marker in close linkage to common bunt resistance genes a valuable tool for marker-assisted selection. Such markers present the possibility of pyramiding several resistance factors into a single wheat line, which may offer a valuable resistance source for organic and low-input agricultural systems. In the present linkage analysis, five SSR markers linked to *Bt9* were identified, as well as a large number of SNP and PAV markers that could be developed into robust markers for *Bt9* selection. In addition, these markers may be useful for future projects aimed at fine mapping the *Bt9* QTL and subsequently identifying the causal gene behind the trait.

### Author contribution statement

The experiment was conceived by PMS, GB, AB and SKR. PMS and AB carried out the phenotypic evaluation. Genotyping with SSR markers as well as analysis of phenotypic data, map construction and QTL analysis was carried out by PMS and AMT, while AMT did the bioinformatics including comparison of genetic and physical maps. The manuscript was drafted by PMS, AMT and SKR, and corrected and approved by all authors.

## Electronic supplementary material

Below is the link to the electronic supplementary material.

Supplementary material 1 (DOCX 29 KB)
