# Supplementary material for: Mapping of common bunt resistance gene Bt9 in wheat
Source: Theor Appl Genet. 2017 Feb 25;130(5):1031–40. doi: 10.1007/s00122-017-2868-6 (PMC5395592; doi:10.1007/s00122-017-2868-6)
Supplement: Supplementary file 1 — Supplementary material 1 (DOCX 29 KB) [file 122_2017_2868_MOESM1_ESM.docx]

**Supplementary Table S1**

List of SSR and Bt10 markers located on chromosome 6D as well as sequences of the DArTseq markers directly flanking the Bt9 gene. For SSR markers and Bt10 specific primers, sequences of the forward and reverse primers, the label used to detect the PCR products and the chromosome location on our map are provided. For DArTseq markers the sequences provided by the company Triticarte Pty. Ltd (Canberra, Australia) are provided. Only the DArTseq markers which can be seen on the Bt9 DH map presented in Fig. 4 are listed

| Marker | Forward Primer (5’-3’) | Reverse Primer(5’-3’) | Label^4)^ | Chromosome location |
| --- | --- | --- | --- | --- |
| **M13-tail** | CACGACGTTGTAAAACGAC |  | 6-FAM, VIC or NED |  |
| **SSR markers^1)^**  BARC202  BARC204  CFD13  CFD42  CFD75  GPW312  GPW362  GPW3087  GPW4005  GPW4372  GPW4510  GPW5125  GPW5179  GPW7303  GPW7433  WMC749  WMC773  WMC822  WMS325  WMS469 | GCGATGTCCAATTTTTCTCCCGTTGC  CGCAGAAGAAAAACCTCGCAGAAAAACC  CCACTAACCAAGCTGCCATT  AGGTTCTAGGGGGCATGTCT  GCATAAACTTGGACCCTGGA  GAACTTTCCACCCCATACCC  GAATCGTCAATGCCCCTCTA  CTCTAAGAAGTCCAATGCAACA  GGTTCACCTCAATAATCGGC  GAGGGAGTAGACATGGTCGC  GTTCCGGTCTCTCTGAGCC  TCCCTATGGTTGCTTCATCC  CCATTCCGCAAATGATGATA  TGCTGTAGATGGCTGTTTCG  GTACATGGAAGAGACCACACCA  GGGTACAGGAGGATCTGACAGG  GAGGCTTGCATGTGCTTGA  CACCCGTCGACCTAGACACC  TTTCTTCTGTCGTTCTCTTCCC  CAACTCAGTGCTCACACAACG | CGATTCGAGTCACGCTGGTA  CGCAGTGTATCCAAATGGGCAAGC  TTTTTGGCATTGATCTGCTG  GCTCTCAATGACTGCACTGG  GCTAAGCCACGCTACCACTC  TTAAGTGAGAAGGAAGCAAAGC  CCCTCTATCGGTCTTTGCTG  AATTGCAGAAACTGGATGCC  GTTCTGGTACGGGTCAATGG  GAACAAAGCGGGAGTGACAT  ATCTGTGACCAGCGGTGAG  GTTGCTTTGAGTCTTTTTGCG  GCGTATTCGGGTTGTTCATT  GCTTTTGCTGACACTTGGAA  CGCTGAGCAAGGACGATAG  TCTCGTCTCCGTCTAGGTTCG  GCCAACTGCAACCGGTACTCT  CGACTGCCCTCTGCTATCCT  TTTTTACGCGTCAACGACG  CGATAACCACTCATCCACACC | M13-tail  M13-tail  M13-tail  M13-tail  M13-tail  M13-tail  M13-tail  M13-tail  M13-tail  M13-tail  M13-tail  M13-tail  M13-tail  M13-tail  M13-tail  M13-tail  M13-tail  M13-tail  TET  M13-tail | 6D  6D  6B, 6D  6D  6D  6A, 6D  6D  6A, 6D  6B, 6D  6D  1B, 6D  6D  6D  6B, 6D  6D  6D, 7B  6D  3A, 3A, 6D  6D  6D |
| **Bt10 PCR^2)^**  Bt10 | GTTTTATCTTTTTATTTC (FSD) | CTCCTCCCCCCA (RSA) | None | 6D |
| **DArTseq ^3)^**  3023416(PAV)  978288(PAV)  1022670(SNP)  3028756(SNP)  1133151(SNP)  3034256(PAV)  3026536(SNP)  1104465(PAV)  1040566(PAV)  3022667(PAV)  1859754(PAV) | TGCAGCCAGAAGACCTAGTTCCCATGGTAAGATTTGCTTCCGAGATCGGAAGAGCGGTTCAGCAGGAAT  TGCAGTGTTGCTGTTTTCTCTCTGTCTGCTCGCATCATCTGTACTAGTATTTCTTATGGCGCCCGAGAT  TGCAGAAATTGTCATGCAAAATTCAGAATGGGGCAGCTCCTCACCAACACGCGGACATTCAAAAAGGGA  TGCAGAGAGCCCTAGGCGTCGTGGGCGACGAGCTCCCCGAGATCGGAAGAGCGGTTCAGCAGGAATGCC  TGCAGCTACCTTTGTCGAGAAACTCGGCGGACACGCGCAGCAGTTCCTCCGAGATCGGAAGAGCGGTTC  TGCAGGTTGGGCAACTTACACAAGACACCAAATAACTGGTCGCCGAGATCGGAAGAGCGGTTCAGCAGG  TGCAGCAGCCGACGTGTCCGAGGGCACGTATGGCACGCCCGAGATCGGAAGAGCGGTTCAGCAGGAATG  TGCAGCGGCACAGAGAAGGAGCCGCGCAATTCCTCACCACGGCACTACGTATCACACGGAGAAGCACAT  TGCAGGTCGCCCTCCTCGTTCCAGATACTAGTGGTAATTTCTGCCGCAGCAAAAGGGGGGCGATTTCAG  TGCAGGCCAGGACCAGGGTTGTTGCTCATGTCCCTGATCCGAGATCGGAAGAGCGGTTCAGCAGGAATG  TGCAGTGGGACACAACGTTCACGGGTGGACAGGTGCAAGCGTTGTGGGACACAATCGTTCAGCGGGAGC | | | 6D  6D  6D  6D  6D  6D  6D  6D  6D  6D  6D |

1. Primer sequences were retrieved from Graingenes (<http://wheat.pw.usda.gov/GG3/>)
2. Primer sequences were retrieved from Laroche et al. (2000).
3. Sequences of DArTseq markers were provided by Triticarte Pty. Ltd (Canberra, Australia)
4. SSR Primers were either directly labelled or labelled with an M13-tail sequence as described in Orabi et al. (2014)

**Supplementary Table S2** Distribution of markers on chromosomes in the *Bt9* DH population. For each chromosome the number of linkage groups assigned to the chromosome, the number of unique positions, the total number of markers and the total length of the linkage groups are shown

| Chromo-some | No of linkage groups | Unique positions | Total no. of markers | Length (cM) |
| --- | --- | --- | --- | --- |
| 1A | 1 | 85 | 423 | 157 |
| 1B | 2 | 105 | 466 | 123 |
| 1D | 1 | 44 | 113 | 119 |
| 2A | 2 | 107 | 532 | 176 |
| 2B | 2 | 185 | 873 | 168 |
| 2D | 1 | 51 | 258 | 141 |
| 3A | 2 | 152 | 452 | 178 |
| 3B | 2 | 121 | 444 | 117 |
| 3D | 1 | 50 | 238 | 122 |
| 4A | 2 | 102 | 397 | 149 |
| 4B | 2 | 72 | 245 | 119 |
| 4D | 1 | 25 | 41 | 104 |
| 5A | 2 | 112 | 378 | 135 |
| 5B | 2 | 74 | 459 | 114 |
| 5D | 2 | 62 | 162 | 168 |
| 6A | 2 | 76 | 349 | 103 |
| 6B | 1 | 27 | 167 | 48 |
| 6D | 1 | 41 | 152 | 139 |
| 7A | 2 | 128 | 575 | 193 |
| 7B | 1 | 48 | 191 | 73 |
| 7D | 2 | 67 | 124 | 236 |
| A genome |  | 762 | 3106 | 1091 |
| B genome |  | 632 | 2845 | 762 |
| D genome |  | 340 | 1125 | 1040 |
| Total |  | 1734 | 7039 | 2882 |
